# Trends in opioid-, alcohol-, and drug-related mortality among US children aged 0–4 years: an analysis of the CDC WONDER Database trends, 1999–2023

**DOI:** 10.1007/s00431-026-07373-9

**Published:** 2026-09-03

**Authors:** Taryn Geiger, Roxana Shahmohammadi Arani, Kennedy Haase, Olivia Anne Foley, Abubakar Tauseef

**Affiliations:** 1 Creighton University School of Medicine, Omaha, NE USA; 2 Department of Medicine, Creighton University School of Medicine, Omaha, NE USA

**Keywords:** CDC WONDER, Opioids, Alcohol, Drugs, Pediatric

## Abstract

Over the past two decades, the USA has observed fluctuating increases in opioid-, alcohol-, and drug-related mortality amongst children under the age of 4. Many factors contribute to mortality, and identifying preventative measures represents a critical gap in public health. This study aims to identify trends in related deaths and highlight potential target areas to combat mortality increases. Data was extracted from the Center for Disease Control and Prevention Wide-ranging Online data for Epidemiologic Research (CDC WONDER) database, and trends in crude mortality rate were analyzed for children ages 0–4 from 1999 to 2023. Results were stratified according to sex, region, race, and age; trends were assessed utilizing Joinpoint analysis software. Overall, crude mortality rates demonstrated an upward trend. Across every census region, there was an increase in mortality, noting the highest in the Midwest region. In nearly every year, rural regions are noted to have a higher crude mortality rate. Male sex was associated with a higher crude mortality rate, and the Non-Hispanic Black or African American population had the largest increase in crude mortality rate compared to other racial/ethnic groups. *Conclusion*: Complex factors contribute to mortality in this population, with clear disparities across geographic locations and demographic groups. These disparities highlight the need for further exploration regarding the effects of the opioid epidemic in the pediatric population. Integrating preventative measures and educational tools into medical education and healthcare systems across the country represents a notable absence in current practice, and direction for future research.

**Supplementary Information:**

The online version contains supplementary material available at https://doi.org/10.1007/s00431-026-07373-9.

## Key messages

***What is known*:**

- Over the past two decades, the USA has observed fluctuating increases in opioid-, alcohol-, and drug-related mortality amongst children under the age of 4.

- Many factors contribute to mortality, and identifying preventative measures represents a critical gap in public health measures and current literature.

***What is new*:**

- Using publicly available epidemiologic data, analysis of mortality stratified by geographic and demographic groupings provides insight into this mortality increase.
- Clear disparities were observed across study subgroups, highlighting the need for collaborative change.

**Supplementary Information:**

The online version contains supplementary material available at https://doi.org/10.1007/s00431-026-07373-9.

## Introduction

Mortality in children aged 0–4 is an important indicator of social health. Despite public health initiatives in recent decades, opioid-, alcohol-, and drug-related morbidities are persistent contributions to pediatric mortality [1]. Substance-related mortality in children aged 0–4 is typically attributed to in utero exposure, postnatal environmental exposure, or accidental ingestion [1]. Substance misuse in parents has increased in recent decades, and with it the risk of child exposure [2]. In 2021, an estimated 500,000 newborns were exposed to illicit drugs or alcohol in utero [3]. According to the Centers for Disease Control and Prevention (CDC), 6 out of every 1000 newborn hospital stays were diagnosed with neonatal abstinence syndrome in 2020 [4]. These mortalities encompass multiple mechanisms such as sudden infant death syndrome (SIDS), drug overdoses, poisonings, and more [5].

Despite wide reaching consequences, a gap in research analyzing opioid-, alcohol-, and drug-related mortality in children aged 0–4 was identified. Previous literature has analyzed mortality across pediatric populations of all ages, with many examining mortality or exposures specific to illicit substances such as fentanyl; however, studies evaluating the impact of all illicit substance exposure on mortality in children aged 0–4 are absent [5, 6]. This study aims to fill this gap and further investigate the impacts of alcohol, opioid, and drug exposure in children during some of their most developmentally vulnerable years.

## Methods

This was a retrospective, population-based ecological study analyzing nationwide mortality data in the USA from 1999 to 2023. The Centers for Disease Control and Prevention Wide-ranging Online Data for Epidemiologic Research (CDC WONDER) and the Multiple Cause-of-Death Public Use Record database death certificate records were utilized to identify drug, opioid, and alcohol as a primary or contributing cause of death on nationwide death certificate records in the USA [1]. Besides opioids and alcohol, substances included drugs, medicaments, and biological substances including, but not limited to, tobacco, narcotics, antibiotics, antiepileptics, psychotropic drugs, and anesthetics [1]. Drug, opioid, and alcohol related mortality were identified using the International Classification of Diseases, 10th Revision, Clinical Modification (ICD-10-CM) codes P96.1, P04.4, P04.3, P04.2, or T36-T50 in patients 0–4 years of age [7]. This age restriction was selected due to the wide range of exposure and vulnerability of this age group to accidental deaths related to these substances. Additionally, ICD-10-CM codes were utilized to capture the breadth of burden across a range of clinical diseases and injuries attributed to opioids, alcohol, and drugs. This study did not require ethical approval as the CDC WONDER database contains anonymized, publicly available data.

Data regarding drug-, opioid-, and alcohol-related deaths and population sizes from 1999 to 2023 were extracted. Inclusion criteria were decedents aged 0 to 4 years at the time of death, deaths occurring between 1999 and 2023, and records where a drug-, opioid-, or alcohol-related condition was documented as either the primary (underlying) or a contributing cause of death (Table 1). Any decedents not meeting all three criteria were excluded by the database itself.

**Table 1:** ICD-10-CM codes and corresponding clinical disease or injury

Clinical modification code	Corresponding clinical disease or injury
P04.2	Newborns affected by maternal use of tobacco, affected by exposure in utero to tobacco smoke. Excluding newborn exposure to environmental tobacco smoke
P04.3	Newborns affected by maternal use of alcohol. Excluding fetal alcohol syndrome
P04.4	Newborns affected by maternal use of drugs of addiction. Includes unspecified drugs of addiction, cocaine, hallucinogens, other drugs of addiction. Excludes maternal anesthesia and analgesia. Excludes withdrawal symptoms from maternal use of drugs of addiction
P96.1	Neonatal withdrawal symptoms from maternal use of drugs of addiction. Excludes reactions and intoxications from maternal opiates and tranquilizers used during labor and delivery
T36-T50	Poisoning by adverse effects of, and underdosing of, drugs, medicaments, and biological substances

Data on demographic and regional groups were stratified, including sex, race/ethnicity, age, urban–rural classification, and region. Racial/ethnic groups were defined as Non-Hispanic White, Non-Hispanic Black or African American, Non-Hispanic American Indian and Alaskan Native, Non-Hispanic Asian or Pacific Islander, and Hispanic or Latino as identified on death certificates. Racial/ethnic groups for Non-Hispanic Asian or Pacific Islander and Non-Hispanic American Indian or Alaska Native were excluded due to suppressed and unreliable data points. Age groups were stratified as 0 to 1 year of age and 1 to 4 years of age. This narrow age restriction was selected due to the distinct exposure profiles and vulnerabilities to accidental substance-related deaths unique to these developmental stages. For urban–rural classifications, the National Center for Health Statistics Urban–Rural Classification Scheme was used to divide the population into urban (large metropolitan area population ≥ 50,000) and rural (population < 50,000) counties per the 2013 United States census classification [8]. Regions were classified into Northeast, Midwest, South, and West according to the Census Bureau definitions [9].

To maintain strict confidentiality and protect against the potential disclosure of personal health information, the CDC WONDER database applies standard data-suppression constraints. All subnational mortality counts representing fewer than 10 deaths (0–9 cases) were automatically suppressed [9]. Consequently, corresponding crude mortality rates and confidence intervals based on these suppressed counts could not be calculated and were excluded from specific subgroup trend profiles. Additionally, in accordance with national vital statistics standards, crude mortality rates calculated from a death count of fewer than 20 cases were flagged as “statistically unreliable” due to high sampling variability [1]. The database output excludes these numerical values due to either suppressed or unreliable designations and were therefore excluded from our analysis completely.

Drug-, opioid-, and alcohol-related crude mortality rates were calculated. Age-adjusted mortality rates were not reported due to the narrow inclusion range of 0–4 years of age. Denominators to calculate these rates vary by year and were derived by the database using year-specific U.S Census population estimates [10, 11]. The Joinpoint Regression Program (Joinpoint version 5.4.0 available from National Cancer Institute, Bethesda, Maryland) was used to determine trends in mortality [12]. This program utilizes data to test significance using the Weighted BIC method [13]. Joinpoints represent a significant change in the data measured. To guarantee model stability and parsimony, the grid search was subject to strict structural constraints: a minimum of 2 observed data points were required between any two consecutive joinpoints, a minimum of 3 observed data points were required between a joinpoint and either end of the time series, and a maximum of 4 joinpoints allowed [13]. The Empirical Quantile (EmpQ) method was chosen in model-selection settings. Unreliable data was deleted from the dataset before uploading to Joinpoint as the software cannot run with non-numerical values. Annual percentage change (APC) and average annual percent change (AAPC) with 95% confidence intervals (CIs) for the crude mortality rate were calculated for the line segments linked to a Joinpoint. APC and AAPCs were considered to increase or decrease if the slope describing the change in mortality over the time interval was significantly different from zero using two-tailed *t* tests. To evaluate the robustness of our trend estimations against years with low event counts or high data volatility, a secondary sensitivity analysis was performed. Models were re-run using a weighted least squares approach, where the variance of the annual crude rates inversely determined the analytical weight of each data point [13]. This down-weighted the influence of unstable annual rates driven by sparse event counts, verifying that the core joinpoint transitions and calculated AAPCs were structurally sound and not artifacts of low-count variance. Statistical significance was set at *p* ≤ 0.05. Asterisks, “*,” denotate statistical significance.

## Results

### Overall

From 1999 to 2023, there were 5786 deaths in children ages 0–4 related to opioids, alcohol, or drugs in the USA. Overall, crude mortality rates increased during this period from 0.62 (95% CI 0.51–0.73) in 1999 to 1.99 (95% CI 1.79 to 2.20) per 100,000 in 2023 with an AAPC of 5.88* (95% CI 5.11 to 6.95) (Supplemental Table 1). The APC in crude mortality rate was 13.05* (95% CI 6.90 to 19.79) from 1999 to 2006, which decreased to 2.81 (95% CI − 1.46 to 6.23) from 2009 to 2019 (Supplemental Fig. 1). The APC later increased to 13.01* (95% CI 7.22 to 23.87) between the years 2019 and 2023 (Supplemental Table 1).

### Demographic differences

#### Sex stratified

**Fig. 1:**
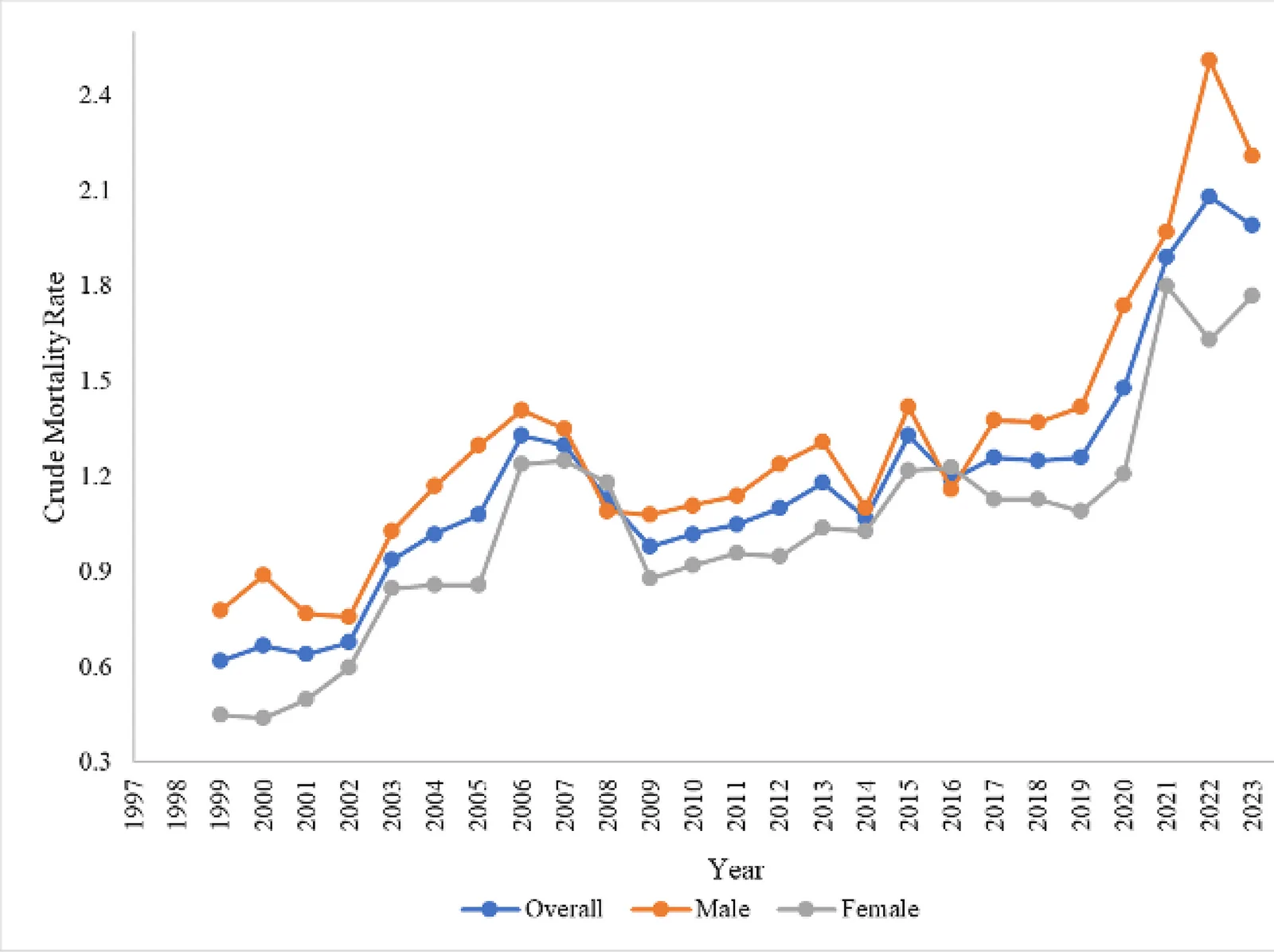
Opioid, alcohol, and drug crude mortality rate per 100,000 people; overall and stratified by sex, 1999–2023

From 1999 to 2023, opioids, alcohol, and drugs contributed to 3273 (56.57%) deaths in males and 2513 (43.43%) in females. Across the duration of the study, the male sex had a higher crude mortality rate in comparison to the female sex (Fig. 1).

The crude mortality rate increased in males from 0.78 (95% CI 0.61 to 0.97) in 1999 to 2.21 (95% CI 1.91 to 2.51) in 2023, with an AAPC of 5.15* (95% CI 4.10 to 7.47) (Fig. 1). The APC in crude mortality rate was 9.55* (95% CI 3.78 to 39.31) from 1999 to 2005, then accelerated to 10.62* (95% CI 5.87 to 26.25) from 2016 to 2023 (Supplemental Fig. 1).

In females, the crude mortality rate increased from 0.45 (95% CI 0.32 to 0.61) in 1999 to 1.77 (95% CI 1.49 to 2.04) in 2023 with an AAPC of 5.98 (95% CI 5.21 to 7.28) (Supplemental Table 1). The APC in crude mortality rate was 15.37* (95% CI 11.87 to 21.87) from 1999 to 2007, later decreasing between 2007 and 2010 at − 12.93* (95% CI − 17.55 to − 1.60) (Supplemental Fig. 1). Between 2010 and 2023, the APC increased to 5.26* (95% CI 3.96 to 7.78) (Supplemental Fig. 1).

#### Race stratified

From 1999 to 2023, opioids, alcohol, and drugs contributed to 1868 (34.55%) deaths in the Non-Hispanic Black or African American population, 2660 (49.20%) deaths in the Non-Hispanic White population, and 879 (16.26%) deaths in the Hispanic or Latino population in the USA.

**Fig. 2:**
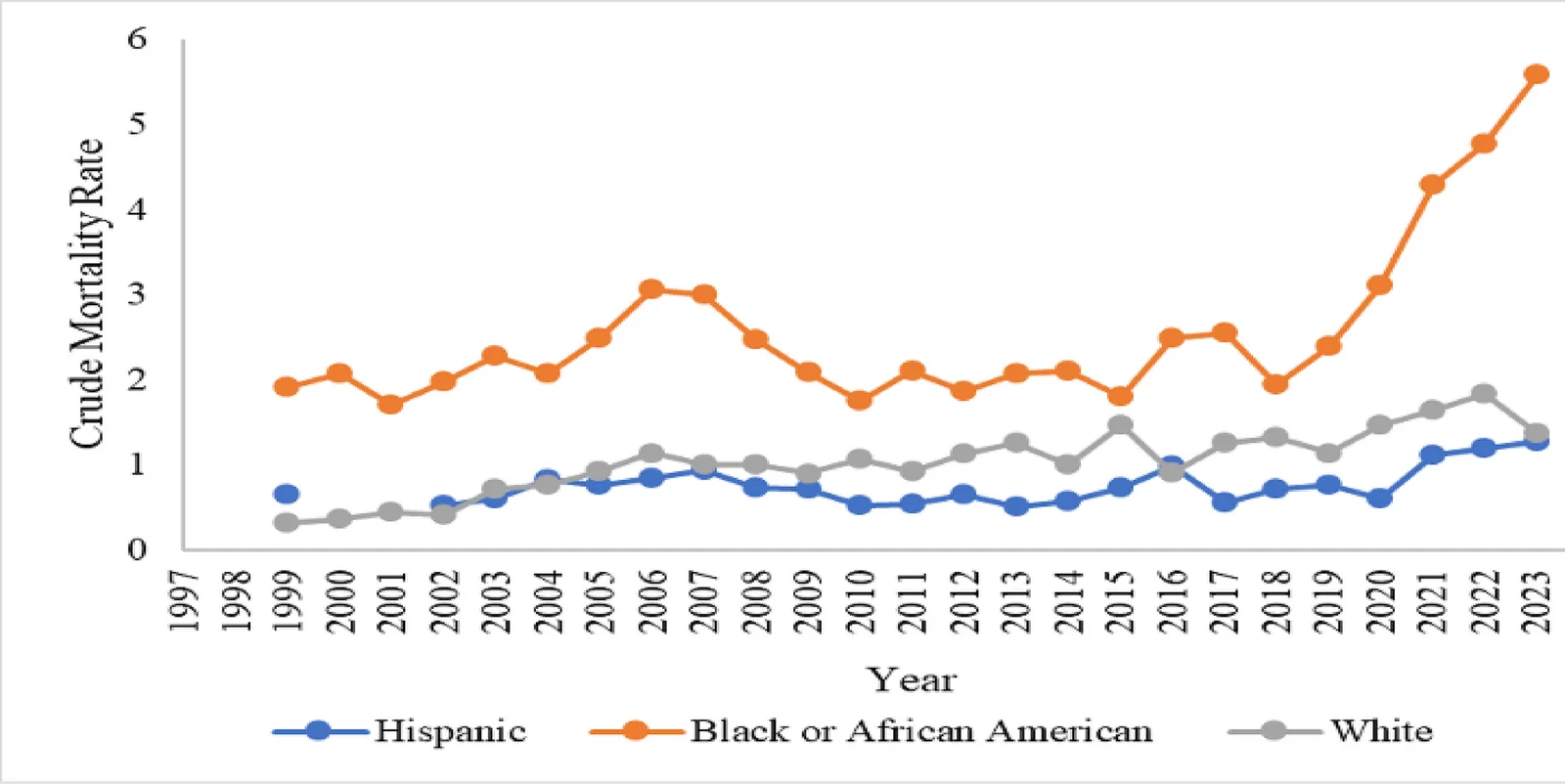
Opioid, alcohol, and drug crude mortality rate per 100,000 people; stratified by race demographics, 1999–2023

The Non-Hispanic Black or African American population had the highest crude mortality rate throughout the study, which increased from 1.92 (95% CI 1.45 to 2.49) in 1999 to 5.59 (95% CI 4.69 to 6.50) in 2023 with an AAPC of 5.19 (95% CI 4.32 to 6.34) (Fig. 2). The APC was 6.68* (95% CI 3.51 to 12.52) from 1999 to 2007, which reduced to − 14.61* (95% CI − 19.57 to − 2.52) from 2007 to 2010 (Supplemental Fig. 2). A small increase was observed from 2010 to 2019 with an APC of 3.46 (− 1.46 to 10.35), with a sharper increase at 24.13* (95% CI 15.53 to 41.73) from 2019 to 2023 (Supplemental Fig. 2).

The Hispanic or Latino population had the lowest crude mortality rate from 1999 to 2023, which increased from 0.66 (95% CI 0.43 to 0.99) in 1999 to 1.28 (95% CI 0.99 to 1.63) in 2023 with an AAPC of 3.01* (95% CI 0.11 to 6.42) (Supplemental Table 2). A steady APC was observed from 1999 to 2020 at 0.24 (95% CI − 7.91 to 28.29), later increasing to 24.62* (95% CI 1.88 to 59.45) from 2020 to 2023 (Supplemental Fig. 2). It is noted that data is not included for the years 2000–2001 due to unreliable designation.

Comparatively, the Non-Hispanic White population had a higher crude mortality rate than Hispanic or Latino population at 0.31 (95% CI 0.22 to 0.43) in 1999 and increasing to 1.37 (95% CI 1.12 to 1.61) in 2023 with an AAPC of 7.10* (95% CI 5.68 to 9.75) (Fig. 2). The APC was 20.54* (95% CI 11.73 to 50.86) from 1999 to 2005. Following, the APC decreased to 2.96* (95% CI 1.26 to 4.31) from 2005 to 2023 (Supplemental Fig. 2).

### Age group differences

From 1999 to 2023, opioids, alcohol, and drugs were linked to 4282 (74.00%) deaths in the < 1 year age group and 1504 deaths (25.99%) in the 1–4 years age in the USA. The population of < 1 year had a consistently higher crude mortality rate compared to the 1–4 age group (Supplemental Table 3).

**Fig. 3:**
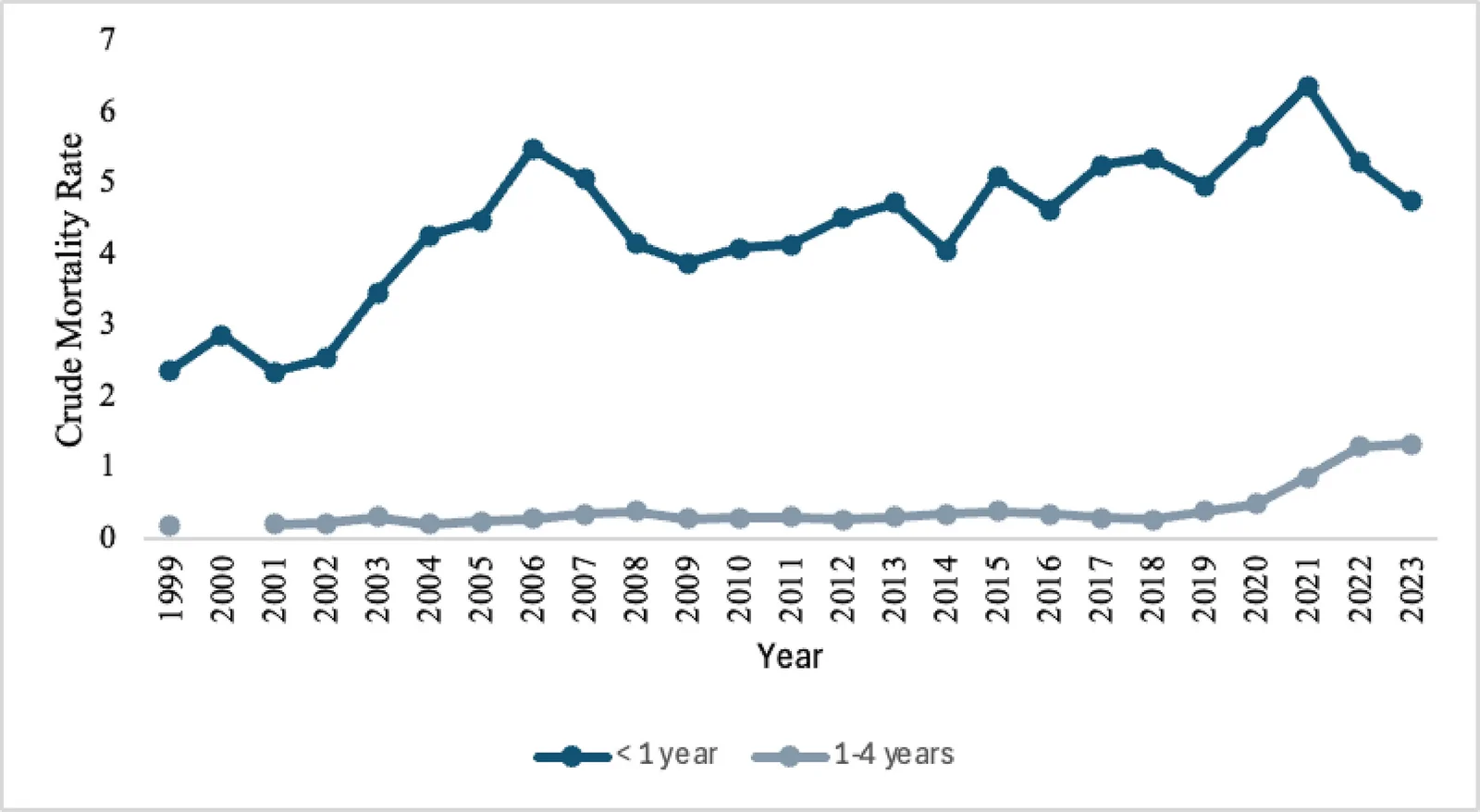
Opioid, alcohol, and drug crude mortality rate per 100,000 people; stratified by age groups, 1999–2023

The crude mortality rate in the < 1 year population increased from 2.37 (95% CI 1.91 to 2.91) in 1999 to 4.74 (95% CI 4.03 to 5.45) in 2023, with an AAPC of 3.38* (95% CI 2.51 to 4.43) (Fig. 3). The < 1 year population had an APC of 13.80* (95% CI 10.15 to 19.00) between the years 1999 and 2006. However, trends flipped between 2006 and 2009, with an APC of − 9.69 (95% CI −13.73 to 0.61) (Supplemental Fig. 3). Trends flipped again between the years 2009 and 2021, with an APC of 3.53* (95% CI 2.34 to 11.20). The APC once again decreased between the years 2021 and 2023, with a value of − 10.31 (95% CI − 19.33 to 1.75). (Supplemental Fig. 3).

The crude mortality rate in the 1–4 years age population increased from 0.18 (95% CI 0.12 to 0.26) in 1999 to 1.32 (95% CI 1.13 to 1.50) in 2023 (Fig. 3). This age group had a significant increase in mortality rates with an AAPC of 8.13* (CI 6.74 to 9.94) throughout the study period (Supplemental Table 3). The 1–4 years population had an APC of 1.99 (95% CI − 0.86 to 4.46) from the years 1999 to 2018. APC then increased to 35.05* (95% CI 23.54 to 62.08) in 2018 to 2023. (Supplemental Fig. 3).

### Regional variations

#### Census region-based differences

**Fig. 4:**
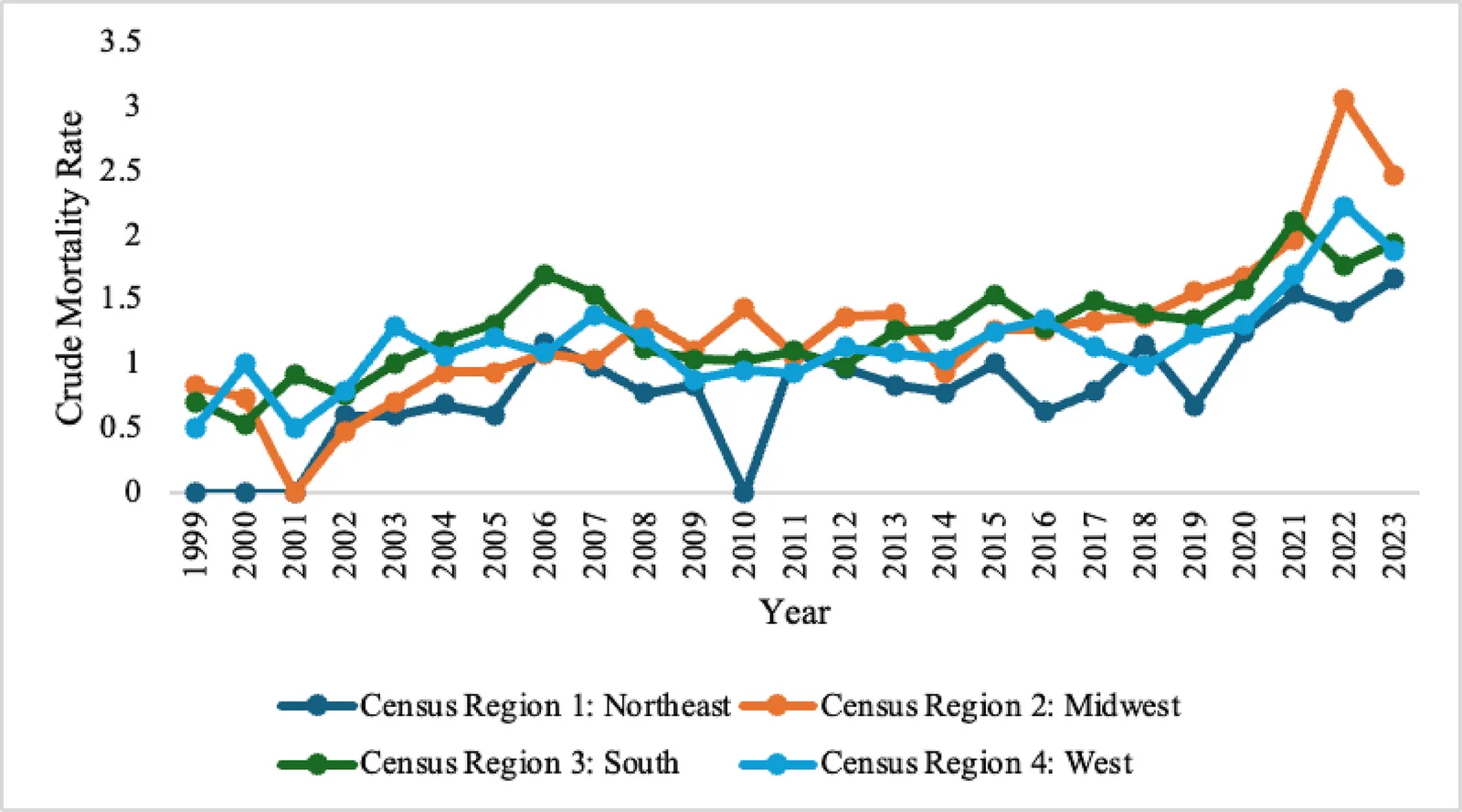
Opioid, alcohol, and drug crude mortality rate per 100,000 people; stratified by census region, 1999–2023

Between 1999 and 2023, opioids, alcohol, and drugs were linked to 692 (11.96%) deaths in the Northeast, 1325 deaths (22.90%) in the Midwest, 2393 (41.36%) deaths in the South, and 1376 (23.78%) deaths in the West (Supplemental Table 4). Each census region saw an increase in crude mortality rate over time, with the Midwest seeing the greatest increase from 0.83 (95% CI 0.58 to 1.15) in 1999 to 2.47 (95% CI 2.00 to 3.20) in 2023 with a consistent AAPC of 5.60* (95% CI 4.16 to 6.93) (Fig. 4). The APC increased from 3.38* (95% CI 0.57 to 4.84) between 1999 and 2019 to 17.42* (95% CI 7.48 to 40.21) between 2019 and 2023 (Supplemental Fig. 4).

In the Northeast, crude mortality rate increased from 0.60 (95% CI 0.37 to 0.92) in 2002 to 1.66 (95% CI 1.23 to 2.19) in 2023, with an APC and AAPC of 3.39* (95% CI 1.71 to 5.29) (Fig. 4). The South saw an increase in crude mortality rate from 0.71 (95% CI 0.52 to 0.94) in 1999 to 1.94 (95% CI 1.63 to 2.26) in 2023, with an AAPC of 5.04* (95% CI 4.21 to 6.43) (Fig. 4). The South also had an APC of 15.54* (95% CI 11.37 to 24.61) between the years 1999 and 2006. The APC decreased to −15.12* (95% CI − 20.20 to − 3.17) between 2006 and 2009, then increased again to 4.83* (95% CI 3.53 to 7.27) between 2009 and 2023 (Supplemental Fig. 4). Lastly, the West had the lowest crude mortality rate in the study period, starting at 0.50 (95% CI 0.32 to 0.75) in 1999 and increasing to 1.88 (95% CI 1.49 to 2.34) in 2023, with an AAPC of 3.69* (95% CI 1.39 to 5.72) (Supplemental Table 4). The Western region had an APC of 1.54 (95% CI − 9.67 to 21.70) between 1999 and 2019, and an APC of 15.13* (95% CI 2.22 to 45.59).

#### Rural vs. urban

From 1999 to 2020, opioids, alcohol, and drugs were linked to 3869 (82.76%) deaths in urban areas, and 806 deaths (17.24%) in rural areas in the USA. When comparing populated regions, crude mortality rates were highest in rural areas compared to urban regions every year in the study besides 2008, which saw a crude mortality rate of 1.16 in urban regions versus 1.00 in rural areas (Supplemental Table 5).

**Fig. 5:**
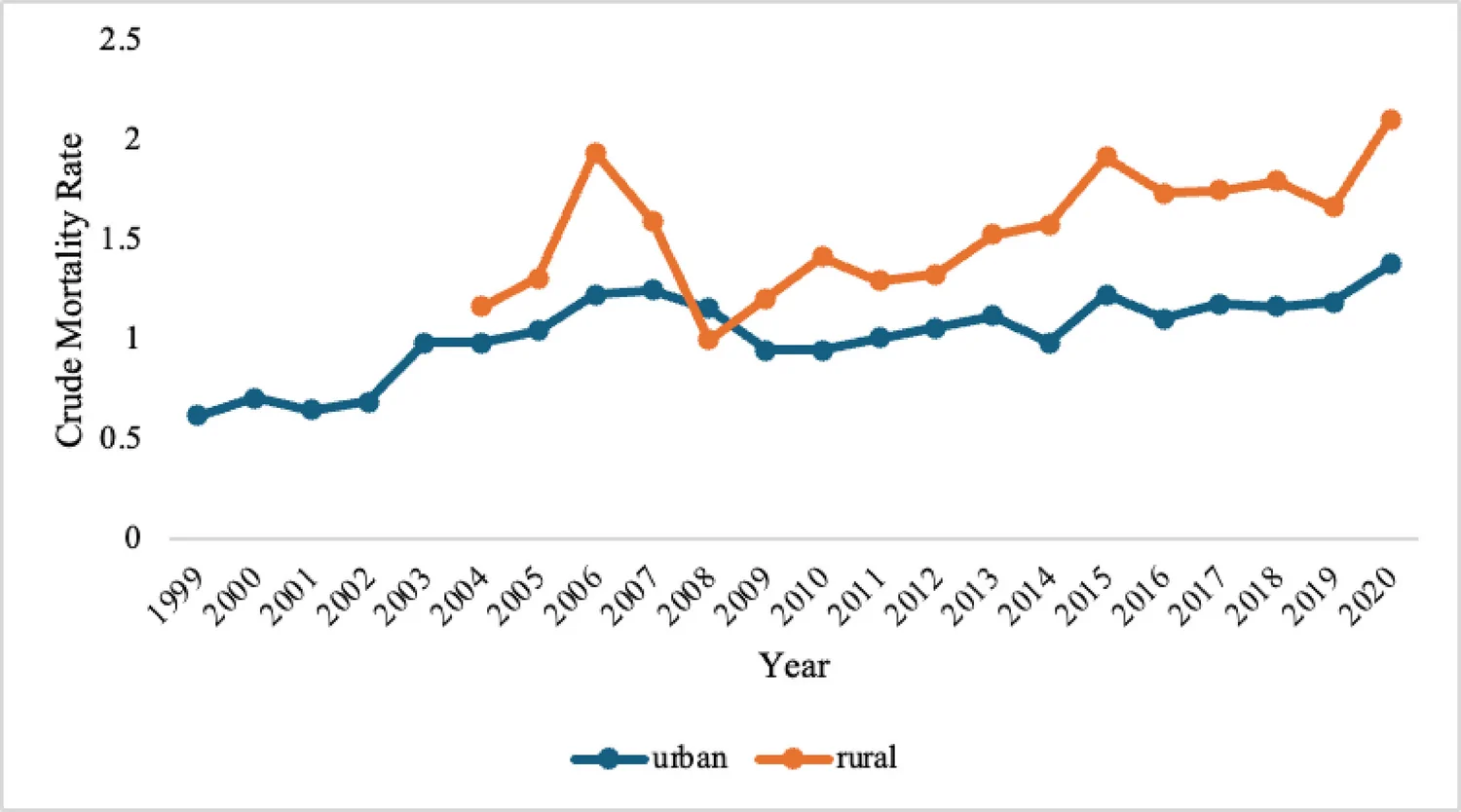
Opioid, alcohol, and drug crude mortality rate per 100,000 people; stratified by rural vs urban, 1999–2020

Rural areas saw crude mortality rates change from 1.17 (95% CI 1.01 to 1.33) in 2004 to 2.11 (95% CI 1.59 to 2.75) in 2020, with a consistent AAPC and APC of 2.31 (95% CI 0.72 to 4.13) (Supplemental Fig. 5). Rual data from the years 1999–2003 was excluded from this study due to unreliable designation. Urban regions had a crude mortality rate of 0.62 (95% CI 0.5 to 0.74) in 1999, which rose to 1.38 (95% CI 1.20 to 1.56) in 2020 (Fig. 5). Urban regions also had a consistent AAPC of 3.76* (95% CI 3.06 to 4.99), with an APC of 9.95* (95% CI 7.18 to 15.15) from 1999 to 2007, − 9.30* (95% CI − 13.41 to − 0.01) from 2007 to 2010, and 3.13* (95% CI 1.28 to 9.89) from 2010 to 2020 (Supplemental Fig. 5).

## Discussion

A significant increase in the crude mortality rate of alcohol, opioid, and drug related mortalities in ages 0–4 across the last three decades can be hypothesized to involve a complex combination of factors. Between 1999 and 2016, opioid-related mortality rates in this age group increased by 225%, rising from 0.08 to 0.26 per 100,000 [5]. This represents one of the largest relative increases across all pediatric age groups. The increase in deaths among young children may reflect broader trends in the opioid epidemic affecting the USA. The overall pediatric opioid mortality rate increased 268% from 1999 to 2016, with nearly 9000 children and adolescents dying from prescription and illicit opioid poisonings during this period [5]. While adolescents aged 15–19 years had the highest absolute rates, the 0–4-year age group experienced the second-largest proportional increase [5]. Crude mortality rate also increased in both age groups around 2019, coinciding with the start of the COVID-19 pandemic. Although this cannot be directly linked to the onset of the COVID-19 pandemic, the increase should be further investigated alongside other explanations such as changes in supply or increased reporting during this period. A cross-sectional study found a 25.6% (95% CI, 13.2%–39.4%) increase in overall ingestions of cannabis, opioids, and alcohol during the pandemic by children under the age of 6 [14]. The increase in ingestions among young children coincided with pandemic-related factors, which may have included increased caregiver substance use, loss of childcare particularly affecting families with children under 5 years, and inadequate substance safekeeping practices [14]. While serious acute adverse drug reactions from medications in breast milk appear to be uncommon overall, infants < 1 year of age (particularly those < 1 month of age) appear most susceptible to adverse effects [15].

Social determinants of health, specifically long-standing structural barriers in access to healthcare, are widely considered key contextual frameworks for understanding demographic disparities. Our results found the highest crude mortality rate among the Non-Hispanic Black or African American population. However, this result contrasts with the trends seen in neonatal opioid withdrawal syndrome (NOWS) across the USA. Current data suggests that NOWS, and resultant complications from in utero opioid exposure, is highest in White Americans [16]. There is limited data surrounding cause of death in children under the age of 4 due to opioid exposure that specifically examines differences among race for infants with NOWS. Future studies examining the age groups, racial, and ethnic classifications of available data could allow for better exploration and identification of these fatal causes of death pertaining to opioid, alcohol, and drugs in young children and infants.

Children under the age of one were noted to have the highest crude mortality rate between age groups. These differences may be related to prenatal exposure to opioids, alcohol, or drugs. Prenatal exposure can substantially increase odds of infant mortality well beyond the neonatal period, especially in children that were not diagnosed with NOWS at birth [17]. Approximately 25% of mortalities under the age of 1 in opioid exposed infants were due to sudden infant death syndrome (SIDS) [17]. Significant data reporting infant fatality from opioids passing through maternal breast milk is lacking; however, further investigation into this phenomenon is of interest in exploring this age-related disparity [18].

Methods to prevent accidental drug and opioid overdose in young children have been implemented across the USA following the 1970 passage of the Poison Prevention Packaging Act. This act aimed to implement safety measures in containers of medications, chemicals, and other common household hazardous substances to prevent accidental deaths in children [19]. Several case studies continue to cite accidental ingestion of a guardian’s medication, or misuse of medications resulting in fatal events of young children [20]. Under the age of one, children are especially vulnerable to accidental ingestion. Drug-related deaths in children aged < 1 also have a higher percentage of drug related mortalities due to homicide at 34.5% compared to children in the 1–4-year group, whose deaths are more likely to be accidental and exploratory [5, 21]. Working to lower rates of opioid prescriptions, which may reduce household drug availability, and ensuring proper guardian education are critical to protecting this vulnerable population.

Regional trends showed an increase in the crude mortality rate in every census region during the study period. The Midwest experienced the largest increase in crude mortality rate, while the Northeast experienced the lowest. Across the USA, the opioid epidemic became a widespread public health concern in 2013, with a decline in recent years. The most recent CDC data on opioid dispensing rates varies widely across counties within states; however, a consistent trend from 2019 to 2023 shows that Southern states consistently have higher rates of opioid prescriptions than Midwest states [22]. Higher rates of opioid prescriptions can be hypothesized as conditions that increase availability of opioids in households and thus possibly contribute to access and accidental ingestion. Regional literature on pediatric mortalities related to opioids, alcohol, and drug is limited. However, analyzing literature studying adult mortality related to drugs, opioid, and alcohol reveals that the Midwest experienced the highest overall increase in drug overdose mortality of all regions (6.0-fold) between the years of 1999 and 2020, compared to a 2.6-fold increase in the Northeast [23]. We hypothesize that these broader regional trends in adult populations potentially influenced environmental conditions—including higher rates of parental substance use, increased drug availability in households, and greater community-level exposure—that potentially affected young children in the Midwest compared to the Northeast. The contrast between opioid prescription rates and adult mortality tied to opioids should be noted as a point of clarification in future studies exploring regional differences. Using these trends, specific investigation into this age group may provide better insight into the contrasting geographic variation noted from our study results.

Results demonstrated a higher crude mortality rate in rural regions in nearly every year of the study period, apart from 2008. This finding is interesting as previous studies have shown that drug overdose death rates in nonmetropolitan areas increased dramatically, and by 2015, slightly surpassed metropolitan rates (17.0 versus 16.2 per 100,000), reversing the historical pattern where urban areas had higher rates [24]. Previous studies have also found that drug poisoning mortality is concentrated in specific rural regions, particularly the Appalachia and parts of the Midwest [25]. It is important to note that rural populations across all racial and ethnic groups experience higher all-cause mortality than urban counterparts, with particularly elevated rates among American Indian or Alaska Native populations for substance-related deaths [26]. However, the lack of age-specific data for the 0–4-year age group in each region limits definitive conclusions and leaves room for further inquiry. Because our macro-level data lacks individual-level clinical metrics, these geographic differences serve as potential exploratory associations rather than direct causal evidence.

## Limitations

This study has several limitations. First, the study relies on data collected from death certificates in the USA, the accuracy of which depends on investigations completed at time of death, meaning common errors in the classification of both cause and manner of death may occur. Second, the available data did not allow for detailed characterization of the circumstances surrounding each death or precise identification of the opioids involved, including differentiation between prescribed and illicitly manufactured fentanyl [27]. Using an ecological study design, limitations arise in linking directly to individuals as only overarching group data is analyzed. Lastly, several years in different stratifications were omitted by the database. Despite these limitations, this study identifies priorities for further research, public health initiatives, and policies that may lead to reduced harm to infants and children.

## Conclusion

Protecting children from alcohol, opioid, and drug-related deaths begins with the collection of well-organized data that allows for identification of target areas where safety, public policy, and healthcare intersect to support change. This study identified a clear increase in crude mortality rate across not only regions but sex and age demographics as well. This highlights the need for over-arching policy changes as opposed to area-targeted implementation. Aiding in educating guardians on methods to safely store substances, warning signs of ingestion, and steps in seeking care provide a foundational idea to build from. Working with an interdisciplinary team, seeking swift and realistic solutions to this persistent problem should be of note across the literature.

## Supplementary Information

Below is the link to the electronic supplementary material.

ESM 1 (DOCX 653 KB)

## Data Availability

The data that supports the findings of this study are publicly available from the Centers for Disease Control and Prevention Wide-ranging Online Data for Epidemiologic Research (CDC WONDER) and the Multiple Cause-of-Death Public Use Record database (https://wonder.cdc.gov). This data is publicly accessible through CDC WONDER online system, and the parameters used to generate this dataset are described in the methods section and data availability statement of this manuscript.

## Abbreviations

APC: Annual percentage change
AAPC: Average annual percent change
CI: Confidence interval
CDC: Centers for Disease Control
CDC WONDER: Centers for Disease Control and Prevention Wide-ranging Online Data for Epidemiologic Research
ICD-10-CM: International Classification of Diseases 10th Revision Clinical Modification
NOWS: Neonatal opioid withdrawal syndrome
SIDS: Sudden infant death syndrome
