# Supplementary material for: Trends in opioid-, alcohol-, and drug-related mortality among US children aged 0–4 years: an analysis of the CDC WONDER Database trends, 1999–2023
Source: Eur J Pediatr. 2026 Sep 3;185(9):717. doi: 10.1007/s00431-026-07373-9 (PMC13541917; doi:10.1007/s00431-026-07373-9)
Supplement: Supplementary file 1 [file 431_2026_7373_MOESM1_ESM.docx]

**SUPPLEMENTAL FIGURES AND TABLES**

**Supplemental Table 1**. Opioids, alcohol, and drug crude mortality rate per 100,000 people; overall and stratified by sex, between 1999-2023 for children ages 0-4 years old

|  | Crude Mortality Rate (per 100,000) | | |
| --- | --- | --- | --- |
| Year | Overall | Male | Female |
| 1999 | 0.62 | 0.78 | 0.45 |
| 2000 | 0.67 | 0.89 | 0.44 |
| 2001 | 0.64 | 0.77 | 0.50 |
| 2002 | 0.68 | 0.76 | 0.60 |
| 2003 | 0.94 | 1.03 | 0.85 |
| 2004 | 1.02 | 1.17 | 0.86 |
| 2005 | 1.08 | 1.30 | 0.86 |
| 2006 | 1.33 | 1.41 | 1.24 |
| 2007 | 1.30 | 1.35 | 1.25 |
| 2008 | 1.13 | 1.09 | 1.18 |
| 2009 | 0.98 | 1.08 | 0.88 |
| 2010 | 1.02 | 1.11 | 0.92 |
| 2011 | 1.05 | 1.14 | 0.96 |
| 2012 | 1.10 | 1.24 | 0.95 |
| 2013 | 1.18 | 1.31 | 1.04 |
| 2014 | 1.07 | 1.10 | 1.03 |
| 2015 | 1.33 | 1.42 | 1.22 |
| 2016 | 1.19 | 1.16 | 1.23 |
| 2017 | 1.26 | 1.38 | 1.13 |
| 2018 | 1.25 | 1.37 | 1.13 |
| 2019 | 1.26 | 1.43 | 1.09 |
| 2020 | 1.48 | 1.74 | 1.21 |
| 2021 | 1.89 | 1.97 | 1.80 |
| 2022 | 2.08 | 2.51 | 1.63 |
| 2023 | 1.99 | 2.21 | 1.77 |
| **Number of Joinpoints (Years of Joinpoint)** | **3 (2006, 2009, 2019)** | **2 (2005, 2016)** | **2 (2007, 2010)** |
| **APC Segment-1 (95% CI)** | 13.05* (6.90 to 19.79) | 9.55* (3.78 to 39.31) | 15.37* (11.87 to 21.87) |
| **APC Segment-2 (95% CI)** | -8.10 (-11.89 to 15.20) | -0.44 (-14.83 to 1.81) | -12.93* (-17.55 to –1.60) |
| **APC Segment-3 (95% CI)** | 2.81 (-1.46 to 6.23) | 10.62* (5.87 to 26.25) | 5.26* (3.96 to 7.78) |
| **APC Segment-4 (95% CI)** | 13.01* (7.22 to 23.87) | -- | -- |
| **Average APC (AAPC) (95% CI)** | 5.88* (5.11 to 6.95) | 5.15* (4.10 to 7.47) | 5.98* (5.21 to 7.28) |

**Supplemental Figure I.** Joinpoint model of opioid, alcohol, and drug related APC overall and stratified by sex, 1999-2023 (*indicates the APC is statistically significant)

**Supplemental Table 2.** Opioids, alcohol, and drug crude mortality rate per 100,000 people; stratified by race demographics, between 1999-2023 for children ages 0-4 years old

|  | Crude Mortality Rate (per 100,000) | | |
| --- | --- | --- | --- |
| Year | Hispanic | Black or African American | White |
| 1999 | 0.66 | 1.92 | 0.31 |
| 2000 | -- | 2.08 | 0.36 |
| 2001 | -- | 1.71 | 0.45 |
| 2002 | 0.52 | 1.98 | 0.41 |
| 2003 | 0.61 | 2.29 | 0.72 |
| 2004 | 0.83 | 2.08 | 0.76 |
| 2005 | 0.76 | 2.49 | 0.93 |
| 2006 | 0.84 | 3.07 | 1.14 |
| 2007 | 0.94 | 3.00 | 1.01 |
| 2008 | 0.74 | 2.48 | 1.00 |
| 2009 | 0.71 | 2.09 | 0.90 |
| 2010 | 0.53 | 1.76 | 1.07 |
| 2011 | 0.54 | 2.10 | 0.93 |
| 2012 | 0.66 | 1.86 | 1.13 |
| 2013 | 0.51 | 2.07 | 1.26 |
| 2014 | 0.58 | 2.10 | 1.01 |
| 2015 | 0.74 | 1.80 | 1.47 |
| 2016 | 0.99 | 2.49 | 0.91 |
| 2017 | 0.56 | 2.55 | 1.26 |
| 2018 | 0.72 | 1.94 | 1.32 |
| 2019 | 0.77 | 2.40 | 1.14 |
| 2020 | 0.60 | 3.12 | 1.46 |
| 2021 | 1.12 | 4.29 | 1.65 |
| 2022 | 1.20 | 4.78 | 1.84 |
| 2023 | 1.28 | 5.59 | 1.37 |
| **Number of Joinpoints (Years of Joinpoint)** | 1 (2020) | 3 (2007, 2010, 2019) | 1 (2005) |
| APC Segment –1 1 (95% CI) | 0.24 (-7.91 to 28.29) | 6.68* (3.51 to 12.52) | 20.54* (11.73 to 50.86) |
| APC Segment –2 1 (95% CI) | 24.62* (1.88 to 59.45) | -14.6* (-19.57 to –2.52) | 2.96* (1.26 to 4.31) |
| APC Segment –3 1 (95% CI) | -- | 3.46 (-1.46 to 10.35) | -- |
| APC Segment –4 1 (95% CI) | -- | 24.13* (15.53 to 41.73) | -- |
| Average APC (AAPC) (95% CI) | 3.01* (0.11 to 6.42) | 5.19* (4.32 to 6.34) | 7.10* (5.68 to 9.75) |

**Supplemental Figure 2.** Joinpoint model of opioid, alcohol, and drug related APC; stratified by race demographics, 1999-2023 (*indicated the APC is statistically significant)

**Supplemental Table 3**. Opioids, alcohol, and drug crude mortality rate per 100,000 people; stratified by age groups, between 1999-2023 for children ages 0-4 years old

|  | Crude Mortality Rate (per 100,000) | |
| --- | --- | --- |
| Year | < 1 Year | 1-4 Years |
| 1999 | 2.37 | 0.18 |
| 2000 | 2.86 | -- |
| 2001 | 2.34 | 0.19 |
| 2002 | 2.53 | 0.21 |
| 2003 | 3.45 | 0.30 |
| 2004 | 4.26 | 0.19 |
| 2005 | 4.47 | 0.23 |
| 2006 | 5.49 | 0.27 |
| 2007 | 5.06 | 0.33 |
| 2008 | 4.14 | 0.37 |
| 2009 | 3.87 | 0.27 |
| 2010 | 4.08 | 0.28 |
| 2011 | 4.13 | 0.29 |
| 2012 | 4.51 | 0.26 |
| 2013 | 4.72 | 0.30 |
| 2014 | 4.05 | 0.33 |
| 2015 | 5.1 | 0.38 |
| 2016 | 4.63 | 0.34 |
| 2017 | 5.25 | 0.28 |
| 2018 | 5.35 | 0.26 |
| 2019 | 4.97 | 0.37 |
| 2020 | 5.65 | 0.48 |
| 2021 | 6.37 | 0.85 |
| 2022 | 5.29 | 1.29 |
| 2023 | 4.74 | 1.32 |
| Number of Joinpoints (Years of Joinpoint) | 3 (2006, 2009, 2021) | 1 (2018) |
| APC Segment-1 (95% CI) | 13.80* (10.15 to 19.00) | 1.99 (-0.86 to 4.46) |
| APC Segment-2 (95% CI) | -9.69 (-13.73 to 0.61) | 35.05* (23.54 to 62.08) |
| APC Segment-3 (95% CI) | 3.53* (2.34 to 11.20) | -- |
| APC Segment-4 (95% CI) | -10.31 (–19.33 to 1.75) | -- |
| Average APC (AAPC) (95% CI) | 3.38* (2.51 to 4.43) | 8.13* (6.74 to 9.94) |

**Supplemental Figure 3.** Joinpoint model of opioid, alcohol, and drug related APC; stratified by age groups, 1999-2023 (*indicated the APC is statistically significant)

**Supplemental Table 4.** Opioids, alcohol, and drug crude mortality rate per 100,000 people; stratified by census region, between 1999-2023 for children ages 0-4 years old

|  | Crude Mortality Rate (per 100,000) | | | |
| --- | --- | --- | --- | --- |
| Year | Census Region 1: Northeast | Census Region 2: Midwest | Census Region 3: South | Census Region 4: West |
| 1999 | -- | 0.83 | 0.71 | 0.5 |
| 2000 | -- | 0.74 | 0.54 | 1.0 |
| 2001 | -- | -- | 0.92 | 0.5 |
| 2002 | 0.60 | 0.48 | 0.76 | 0.79 |
| 2003 | 0.60 | 0.71 | 1.0 | 1.29 |
| 2004 | 0.69 | 0.94 | 1.18 | 1.07 |
| 2005 | 0.61 | 0.94 | 1.31 | 1.2 |
| 2006 | 1.17 | 1.08 | 1.7 | 1.09 |
| 2007 | 0.98 | 1.03 | 1.54 | 1.38 |
| 2008 | 0.77 | 1.35 | 1.12 | 1.2 |
| 2009 | 0.84 | 1.11 | 1.04 | 0.88 |
| 2010 |  | 1.43 | 1.03 | 0.95 |
| 2011 | 1.09 | 1.07 | 1.1 | 0.93 |
| 2012 | 0.97 | 1.37 | 0.98 | 1.13 |
| 2013 | 0.84 | 1.39 | 1.26 | 1.09 |
| 2014 | 0.78 | 0.93 | 1.27 | 1.04 |
| 2015 | 1.0 | 1.27 | 1.54 | 1.25 |
| 2016 | 0.63 | 1.27 | 1.28 | 1.35 |
| 2017 | 0.79 | 1.34 | 1.49 | 1.13 |
| 2018 | 1.15 | 1.37 | 1.39 | 0.99 |
| 2019 | 0.68 | 1.56 | 1.35 | 1.23 |
| 2020 | 1.25 | 1.68 | 1.58 | 1.3 |
| 2021 | 1.55 | 1.97 | 2.11 | 1.69 |
| 2022 | 1.41 | 3.05 | 1.76 | 2.23 |
| 2023 | 1.66 | 2.47 | 1.94 | 1.88 |
| **Number of Joinpoints (Years of Joinpoint)** | 0 | 1 (2019) | 2 (2006, 2009) | 1 (2019) |
| APC Segment-1 (95% CI) | 3.39* (1.71 to 5.29) | 3.38* (0.57 to 4.84) | 15.54* (11.37 to 24.61) | 1.54 (-9.67 to 21.70) |
| APC Segment-2 (95% CI) | -- | 17.42* (7.48 to 40.21) | -15.12* (-20.20 to –3.17) | 15.13* (2.22 to 45.59) |
| APC Segment-3 (95% CI) | -- | -- | 4.83* (3.53 to 7.27) | -- |
| Average APC (AAPC) (95% CI) | 3.39* (1.71 to 5.29) | 5.60* (4.16 to 6.93) | 5.04* (4.21 to 6.43) | 3.69* (1.39 to 5.72) |

**Supplemental Figure 4.** Joinpoint model of opioid, alcohol, and drug related APC; stratified by census regions, 1999-2023 (*indicated the APC is statistically significant)

**Supplemental Table 5.** Opioids, alcohol, and drug crude mortality rate per 100,000 people; stratified by rural vs urban, between 1999-2020 for children ages 0-4 years old

|  | **Crude Mortality Rate (per 100,000)** | |
| --- | --- | --- |
| **Year** | **Urban** | **Rural** |
| 1999 | 0.62 | -- |
| 2000 | 0.71 | -- |
| 2001 | 0.65 | -- |
| 2002 | 0.69 | -- |
| 2003 | 0.99 | -- |
| 2004 | 0.99 | 1.17 |
| 2005 | 1.05 | 1.31 |
| 2006 | 1.23 | 1.94 |
| 2007 | 1.25 | 1.6 |
| 2008 | 1.16 | 1 |
| 2009 | 0.95 | 1.21 |
| 2010 | 0.95 | 1.42 |
| 2011 | 1.01 | 1.3 |
| 2012 | 1.06 | 1.33 |
| 2013 | 1.12 | 1.53 |
| 2014 | 0.99 | 1.58 |
| 2015 | 1.23 | 1.92 |
| 2016 | 1.11 | 1.74 |
| 2017 | 1.18 | 1.75 |
| 2018 | 1.17 | 1.8 |
| 2019 | 1.19 | 1.67 |
| 2020 | 1.38 | 2.11 |
| Number of Joinpoints (Years of Joinpoint) | 2 (2007, 2010) | 0 |
| APC Segment-1 (95% CI) | 9.95* (7.18 to 15.15) | 2.31* (0.72 to 4.13) |
| APC Segment-2 (95% CI) | -9.30* (-13.41 to –0.01) | -- |
| APC Segment-3 (95% CI) | 3.13* (1.28 to 9.89) | -- |
| Average APC (AAPC) (95% CI) | 3.76* (3.06 to 4.99) | 2.31* (0.72 to 4.13) |

**Supplemental Figure 5.** Joinpoint model of opioid, alcohol, and drug related APC; stratified by rural vs urban, 1999-2020 (*indicated the APC is statistically significant)
